# Elucidating the phylodynamics of endemic rabies virus in eastern Africa using whole-genome sequencing

**DOI:** 10.1093/ve/vev011

**Published:** 2015-09-10

**Authors:** Kirstyn Brunker, Denise A Marston, Daniel L Horton, Sarah Cleaveland, Anthony R Fooks, Rudovick Kazwala, Chanasa Ngeleja, Tiziana Lembo, Maganga Sambo, Zacharia J Mtema, Lwitiko Sikana, Gavin Wilkie, Roman Biek, Katie Hampson

**Affiliations:** ^1^Institute of Biodiversity, Animal Health and Comparative Medicine, University of Glasgow, Glasgow G12 8QQ, UK; ^2^The Boyd Orr Centre for Population and Ecosystem Health, University of Glasgow, Glasgow G12 8QQ, UK; ^3^Animal and Plant Health Agency, Weybridge, Woodham Lane, KT15 3NB, UK; ^4^School of Veterinary Medicine, University of Surrey, Guildford GU2 7XH, UK; ^5^Department of Veterinary Medicine and Public Health, Sokoine University of Agriculture, Morogoro, United Republic of Tanzania; ^6^Tanzania Veterinary Laboratory Agency, Dar es Salaam, United Republic of Tanzania, Temeke Veterinary, Mandela Road, P.O. BOX 9254; ^7^Ifakara Health Institute, Ifakara, United Republic of Tanzania, P.O. Box 53; ^8^MRC Centre for Virus Research, University of Glasgow, Sir Michael Stoker Building, Garscube Campus, 464 Bearsden Road, Glasgow G61 1QH, UK

**Keywords:** RNA virus, phylodynamics, zoonoses, endemic, rabies, translocation

## Abstract

Many of the pathogens perceived to pose the greatest risk to humans are viral zoonoses, responsible for a range of emerging and endemic infectious diseases. Phylogeography is a useful tool to understand the processes that give rise to spatial patterns and drive dynamics in virus populations. Increasingly, whole-genome information is being used to uncover these patterns, but the limits of phylogenetic resolution that can be achieved with this are unclear. Here, whole-genome variation was used to uncover fine-scale population structure in endemic canine rabies virus circulating in Tanzania. This is the first whole-genome population study of rabies virus and the first comprehensive phylogenetic analysis of rabies virus in East Africa, providing important insights into rabies transmission in an endemic system. In addition, sub-continental scale patterns of population structure were identified using partial gene data and used to determine population structure at larger spatial scales in Africa. While rabies virus has a defined spatial structure at large scales, increasingly frequent levels of admixture were observed at regional and local levels. Discrete phylogeographic analysis revealed long-distance dispersal within Tanzania, which could be attributed to human-mediated movement, and we found evidence of multiple persistent, co-circulating lineages at a very local scale in a single district, despite on-going mass dog vaccination campaigns. This may reflect the wider endemic circulation of these lineages over several decades alongside increased admixture due to human-mediated introductions. These data indicate that successful rabies control in Tanzania could be established at a national level, since most dispersal appears to be restricted within the confines of country borders but some coordination with neighbouring countries may be required to limit transboundary movements. Evidence of complex patterns of rabies circulation within Tanzania necessitates the use of whole-genome sequencing to delineate finer scale population structure that can that can guide interventions, such as the spatial scale and design of dog vaccination campaigns and dog movement controls to achieve and maintain freedom from disease.

## 1 Introduction

The general trend of increasing incidence and expansion of emerging or re-emerging zoonotic diseases (e.g., Ebola, Chikungunya, and avian influenza) (Woolhouse 2002; Greger 2007; Jones et al. 2008) and persistence of established zoonoses, such as canine rabies, highlights the ongoing challenges faced as we attempt to characterize and control them. The processes that drive the spread and persistence of infectious diseases are reflected in a genetic signature in pathogen genomes (Biek and Real 2010). Understanding the processes that give rise to spatial population structure in pathogens can inform the management and control of infectious diseases. For example, analyses of evolutionary, epidemiological, and ecological data have recently demonstrated that global live swine trade strongly predicts the global dissemination of influenza A viruses in swine (Nelson et al. 2015), and air travel has been revealed as a major factor driving the intra-continental spread of Dengue virus (Nunes et al. 2014). Viral pathogens, particularly fast-evolving RNA viruses, are model systems to explore pathogen populations as they rapidly accumulate genetic diversity on a timescale similar to epidemiological processes (Drummond et al. 2003; Biek et al. 2015). Statistical phylogeographic approaches are available (Lemey et al. 2009; Bedford et al. 2014; Bielejec et al. 2014) to develop a quantitative understanding of the processes that give rise to spatial patterns in RNA viruses (Holmes and Grenfell 2009) on epidemiological time scales. Whole-genome sequencing (WGS) is increasingly being used as a means to extract these patterns, but it is unclear how much resolution can be gained and at what temporal and spatial scale. In this article, canine rabies virus (RABV) is used as a model to determine the spatio-temporal patterns of an endemic zoonotic virus using whole-genome data to distinguish structure at an increasingly fine scale.

Rabies is a globally distributed zoonotic disease caused by a single-stranded negative sense RNA virus from the *Lyssavirus* genus. Though capable of infecting any mammal, given virus variants are typically maintained in distinct species-specific cycles within the orders Carnivora and Chiroptera (Rupprecht, Hanlon, and Hemachudha 2002). The disease causes thousands of human deaths every year, predominantly in Asia and Africa where the virus circulates endemically in domestic dogs (*Canis lupus familiaris*) (Knobel et al. 2005; Shwiff, Hampson, and Anderson 2013). The majority of these deaths (∼99%) are caused by bites from rabid dogs, instilling fear into the many communities that live under continuous threat from a disease that is almost invariably fatal but entirely preventable. Although the role of domestic dogs as key vectors of rabies is recognized, much less is known about the dog-associated RABV variant than wildlife variants such as raccoon or skunk RABVs circulating in North America (Brunker et al. 2012). Moreover, while epidemic expansions of wildlife RABV have been well documented and studied (e.g., Real et al. 2005; Biek et al. 2007; Kuzmina et al. 2013), we know little about the persistence and spread of rabies in endemic landscapes.

Characterizing the spatial scales of canine rabies dispersal is a critical step toward identifying the processes and factors driving its dynamics and the scale at which control strategies need to be implemented. On a global scale, canine RABV exhibits a strong phylogeographic structure with the distribution of seven distinct major clades reflecting the position of major barriers, such as oceans and mountain ranges, or historical mass human colonization/migration events (David et al. 2007; Bourhy et al. 2008). However, it is unclear whether this genetic structure will persist in endemic scenarios or at smaller scales, and how much it is influenced by human-mediated dispersal. Indeed, on a regional scale, this landscape structure becomes less distinct: some landscape features, for example, geopolitical boundaries can act as apparent barriers to movement, as seen in North Africa (Talbi et al. 2010), whilst contradictory patterns of synchronous cycles of RABV across multiple countries (Hampson et al. 2007) and repeated cross-border incursions (Hayman et al. 2011) have also been observed elsewhere in the continent.

While there is a growing understanding of the epidemiology of canine rabies in Africa (Hampson et al. 2007, 2009; Lembo et al. 2008), effective rabies control is still hindered by limited knowledge of some of the key drivers of viral transmission and spread. Mass dog vaccination is the mainstay of successful rabies control but requires sustained coverage of at least 70 per cent (WHO 2005; Townsend et al. 2013). In addition, spatial heterogeneity may affect how vaccine is most effectively distributed to interrupt key transmission corridors and target regions seeding RABV dispersal. An important aspect of this heterogeneity is the impact of human factors on RABV transmission, which has direct implications for control, including the design and scale of interventions necessary to interrupt transmission and maintain freedom from disease. For example, movement of people between urban and rural areas and the dog meat trade have been postulated as means of spreading RABV through human-mediated dog movements in rabies-endemic countries in Asia (Denduangboripant et al. 2005; Tao et al. 2009; Ahmed et al. 2015). Uncovering the viral population structure and dynamics of RABV in Tanzania may identify similar patterns attributed to human-mediated movements that can aid the identification of sources key to viral persistence.

There have been few in-depth spatial epidemiology studies of RABV in sub-Saharan Africa, probably owing to the lack of resources for effective surveillance including sample collection (Nel 2013). However, recent studies provide intriguing insights into the dynamics of rabies in certain parts of Africa (see Talbi et al. 2010; Mollentze et al. 2014), indicating a degree of spatial structure with evidence of long distance movements facilitated by humans. RABV spatiotemporal dynamics in East Africa in particular are poorly resolved and very few sequences are publically available. At present, little is known about the genetic diversity or structure of RABV in Tanzania other than coarse phylogenetic analyses of partial or full nucleoprotein gene (N gene) sequences, limited to well-studied regions (Kissi, Tordo, and Bourhy 1995; Lembo et al. 2007). Furthermore, whole-genome population studies of RABV have not yet been attempted despite their great potential to provide a better understanding of the processes determining rabies spread and persistence.

In this study, finely resolved space-time-genetic data, including whole-genome sequences, were used to determine the spatial and temporal dynamics of endemic RABV at both a regional and local scale in Tanzania. The utility of partial genome data was also demonstrated as a means to characterize large-scale phylogeographic patterns of RABV in Africa. Specifically, we aimed to characterize the dynamics of rabies virus in an endemic system including the role of human-mediated transport.

## 2 Materials and methods

### 2.1 Samples

For this study, fifty-nine new whole-genome sequences were obtained from animal hosts (primarily domestic dogs) from nine regions in Tanzania sampled between 2003 and 2012. A previously sequenced sample (RV2772; accession: KF155002) (Marston et al. 2013) from Southern Tanzania was included in the dataset and used as a reference sequence (sample details in Supplementary Table S1). The main study area encompassed the Serengeti District in northwest Tanzania where approximately half the samples (*n* = 33) were obtained from active surveillance, which enabled the collection of brain material from suspect rabid animals and GPS coordinates and dates recorded as described in Hampson et al. (2009). The remaining twenty-seven samples were obtained opportunistically from other regions in Tanzania as part of surveillance by the Tanzanian Ministry of Livestock and Fisheries Development and the Tanzanian Veterinary Laboratory Agency. All samples were sent to the Animal & Plant Health Agency (APHA) in Weybridge, UK, for processing.

In addition, fifty new partial N gene sequences (405 bp) from Tanzania were obtained via reverse transcription-polymerase chain reaction (RT-PCR) and Sanger sequencing using samples archived at APHA and submitted to GenBank with accession numbers KR534217–KR534266 (details in Supplementary Material and Table S2).

### 2.2 RNA extraction and WGS

Total RNA was extracted at APHA directly from brain tissue using TRIZOL, according to manufacturer’s instructions (Invitrogen). Precipitated total RNA was re-suspended in molecular-grade water at a one in ten dilution and quantified using a NanoDrop spectrophotometer (Thermo Scientific). Samples were sequenced on a range of next generation sequencing platforms during NGS protocol optimization (see Supplementary Material for details). The majority of samples (*n* = 48) were sequenced by the following method: TRIzol-extracted viral RNA was depleted of host genomic DNA using the on-column DNase treatment in RNeasy plus mini kit (Qiagen) as per manufacturer’s instructions with elution in 30 µl molecular grade water. This was followed by host ribosomal RNA depletion using Terminator 5’-phosphate-dependent exonuclease (Epicentre Biotechnologies), as detailed in (Marston et al. 2013). First- and second-strand cDNA was synthesized using a Roche cDNA synthesis system kit with random hexamers (Roche). Resultant cDNA was quantified using Picogreen dsDNA quantitation reagent (Invitrogen) and approximately 1 ng of each sample used in a ‘tagmentation’ reaction mix using a Nextera XT sample preparation kit (Illumina), according to the manufacturer’s protocol (minus the bead normalization step). DNA libraries for each sample were quantified using a Quant-iT PicoGreen dsDNA Assay Kit (Invitrogen) or a Qubit assay kit (Life technologies), and average library size was measured with a high-sensitivity DNA Bioanalyzer chip on a model 2100 Bioanalyzer (Agilent). Sample libraries were transported to the MRC Centre for Virus Research at the University of Glasgow, UK, for the final steps of library preparation and sequencing. Individual libraries were pooled and normalized to equimolar concentrations at a suitable plexity (x24 for MiSeq runs). Libraries were sequenced as 150-bp paired-end reads on an Illumina MiSeq. Additional sequencing was conducted on a NextSeq 500 platform (Glasgow Polyomics at the University of Glasgow, Glasgow, UK) and reads merged with MiSeq reads to increase coverage for poorly sequenced samples (see Supplementary Material).

### 2.3 Bioinformatics and sequence analysis

Raw reads were assessed in FastQC (Andrews 2010), and Trimmomatic (Bolger, Lohse, and Usadel 2014) was used to trim 3’-ends, remove adapter contamination, and to filter based on quality with default parameters. Filtered reads were mapped to the previously sequenced genome of Tanzanian RABV sample RV2772 (accession: KF155002) with BWA mem version 0.7.10 (Li and Durbin 2009) and converted to bam file format using SAMtools v. 0.1.18 (Li et al. 2009).

A conservative single-nucleotide polymorphism (SNP) calling routine was implemented in GATK utilizing the UnifiedGenotyper tool to identify high confidence SNPs, which had passed GATK filters on strand bias (FS > 60, SOR > 4), mapping quality (MQ < 40, MQRankSum < −12.5), read position (ReadPosRankSum < −8), and depth of coverage (DP < 5). Indels were filtered if FS > 200 and ReadPosRankSum < −20 and further manually inspected for inclusion (e.g., dismissed if near a homopolymer run). Consensus sequences were built using a custom script in R, which called filtered SNPs with a 75 per cent consensus rule (positions with <75% consensus were given a IUPAC code for the corresponding ambiguous base call) and genome positions with a depth of coverage less than two were labelled ‘N’. Potential SNP calls that failed only the depth filter, that is, had a depth < 5 but >1, were passed if the same polymorphism had been present as a high confidence SNP in at least two other samples. Otherwise, the position was given an IUPAC code representing the population-level calls and the potential SNP. In addition, a set of consensus sequences using a more relaxed approach to SNP calling was produced which involved strict calls of all SNPs with depth > 1 and gaps filled with the majority population consensus sequence. These relaxed consensus sequences were used to produce initial starting trees for BEAST analyses (see Section 2.6).

Sequencing resulted in 93–100 per cent coverage of the genome, with >99 per cent genome coverage achieved for 95 per cent of samples and a median depth of coverage of seventy-seven (range: 6–1,871, see Supplementary Table S1). Nextera XT is a transposase-based method of library preparation and sequence reads typically miss the ends of the genome; however, as the ends of lyssaviruses are highly conserved (Marston et al. 2007; Kuzmin et al. 2008), it is unlikely that any informative variation was missed. We, therefore, consider our analyses to be based on genome-wide variation and henceforth refer to our dataset as whole-genome sequences. Consensus sequences were aligned using MAFFT v7.149b (Katoh and Standley 2013) and submitted to GenBank (accession numbers: KR906734–KR906792).

### 2.4 Phylogenetic reconstruction

Initial datasets of (1) partial N gene 405 bp (1,317 sequences) and (2) full N gene 1,350 bp (674 sequences) sequences isolated in Africa were constructed using sequences retrieved from GenBank and including new Tanzanian isolates sequenced for this study (59 new WGS samples and 50 new partial genome sequences). Following maximum likelihood (ML) phylogenetic reconstruction with the initial sequence datasets, subset trees were extracted for samples in the Africa 1b clade (430 samples in the partial N dataset and 100 in the full N dataset).

Alignments for whole genome, full N, and partial N gene were created in MAFFT (Katoh and Standley 2013) and estimated phylogenetic relationships using both ML and Bayesian methods. ML phylogenies were estimated in RAxML (Stamatakis et al. 2012) with a general time reversible (GTR) nucleotide substitution model and a gamma distribution model of among-site rate variation. A Chinese dog RABV sequence from GenBank (accession no: FJ712193) was used as an outgroup, and node support was evaluated with 1,000 bootstrap replicates. Bayesian phylogenetic reconstruction was conducted in BEAST v1.8.1 (Drummond et al. 2012) using a posterior distribution of trees (without a molecular clock model). Phylogenies were visualized and annotated in R using the packages adegenet (Jombart 2008) and APE (Paradis, Claude, and Strimmer 2004), and maps were made in R with Maptools (Lewin-koh et al. 2009) and sp packages (Pebesma and Bivand 2005; Bivand, Pebesma, and Gomez-Rubio 2008). The degree of spatial admixture at large phylogeographic scales, i.e. sub-continental and country level, was quantified by an association index (AI) using BaTS software with beast phylogenies (Parker et al. 2008).

### 2.5 Selecting an evolutionary model

An initial nucleotide substitution model was chosen based on the model selected by PartitionFinder (Lanfear et al. 2012). Our whole-genome alignments were partitioned into sixteen sets of nucleotides: one for each codon position (CP) in each gene (five genes) and one for concatenated non-coding regions. Model scheme selection was based on the best AIC score from a greedy search of substitution models, which favoured a GTR model with partitioning into three CPs (CP123).

In addition, model comparison based on marginal likelihood estimates using path sampling (PS) and stepping stone (SS) sampling implemented in BEAST (100 path steps and a chain length of 100,000 steps) were used to test varying levels of complexity in the substitution model. Non-coding sequence was concatenated and partitioned as a ‘gene’ with its own evolutionary model. Specifically, we tested the HKY model with: (1) a gene-specific nucleotide model with gene-specific rate variation; (2) a gene-linked CP partitioned model with among CP rate heterogeneity and homogeneous rates among genes; and (3) a gene-specific CP partitioned model with among CP and among gene rate heterogeneity. Model types 2 and 3 were also tested with CP112 and CP123 partitioning schemes. Following the results for the best HKY model, we did a final step comparing the most supported HKY model with the same structure but using a GTR model. Results (Supplementary Table S2) strongly favoured GTR and CP models (CP123 was best supported), but there was no support for partitions according to genes, which all had similar rates of substitution. This significantly reduced the complexity of the model and is an important finding in the context of analysing RABV whole-genome sequence.

### 2.6 Bayesian evolutionary analyses

Bayesian Markov-chain Monte Carlo analyses were performed using BEAST v1.8.1 (Drummond et al. 2012) and the BEAGLE library (Ayres et al. 2012). Based on model comparisons, the most supported evolutionary model was a GTR model with different substitution parameters for CPs one, two, and three (GTR_123_ + CP_123_ + Γ_123_) and homogeneous rates among genes, with a GTR + G substitution model for non-coding sequence. A relaxed molecular clock with a lognormal distribution was used to model rate variation among branches with a Bayesian skyline model (Drummond et al. 2005) with ten groups as a flexible tree prior. A Bayesian skyline plot was used to estimate the viral effective population size through time (Drummond et al. 2005). To reconstruct the spatial dynamics of RABV spread in Tanzania, we implemented a discretized diffusion process among nine regional sampling locations, formalized as an asymmetric continuous time Markov chain (CTMC) model (Lemey et al. 2009). Three independent Markov-chain Monte Carlo chains with 50 million states and a sampling frequency of 50,000 were combined in LogCombiner after discarding at least 10 per cent burn. Posterior distributions were inspected in Tracer v1.6 (Rambaut et al. 2014) to ensure adequate mixing and convergence. Initial analyses revealed issues with tree likelihood convergence. Therefore, a CTMC model using relaxed consensus sequences (see above), which contained fewer ambiguities, was implemented first and the maximum clade credibility (MCC) tree used as a starting tree for the final CTMC models with conservative consensus sequences.

To estimate the most significant pathways of viral dispersal between regions, a stochastic search variable selection (BSSVS) procedure was implemented to identify the best supported diffusion rates through Bayes factor (BF) testing (Lemey et al. 2009; Bielejec et al. 2011). For the per lineage rate of migration (Kühnert, Wu, and Drummond 2011), a conditional reference prior (Ferreira and Suchard 2008) is commonly used but for our data resulted in convergence problems with some of the BSSVS parameters. Instead, we used an exponential prior with a mean of 0.01, which gave the most robust results out of a range of values tested (data not shown). The degree of spatial admixture was scored using a modified Association Index (Wang et al. 2001; Lemey et al. 2009) and quantified using the inferred number of lineage migration/movement events between locations estimated by Markov jump counts (Minin and Suchard 2008) along the branches of the posterior tree distribution. For BEAST models with Markov Jump counts implemented, a conditional reference prior on the per lineage rate of migration was chosen. A summarized history of Markov jump counts was used to identify movements between regions that occurred on very short branches and thus over unusually short time frames. Dogs rarely move 1 km from their homestead (Hampson et al. 2009; Woodroffe and Donnelly 2011), and Hampson et al. (2009) found a maximum distance of 20 km between linked RABV cases in the Serengeti District. Lineage migrations between regions, representing distances >100 km, were therefore considered unlikely to be attributable to dog movement alone if they occurred over a period of 2 years or less and were instead interpreted as being the result of human-mediated movement. In addition, the same form of discretized diffusion model was used to assess diffusion at a broader scale, that is, between the North and South of Tanzania, with Pemba Island classed as a third discrete state. A BF test in the program SPREAD (Bielejec et al. 2011) was used to identify well-supported migration pathways (BF > 3). Sampled trees were summarized as an MCC tree with median node heights using TreeAnnotator v.1.8.1, and Figtree v1.4.2 was used to visualize trees and the inferred ancestral locations for internal branches.

## 3 Results

### 3.1 Geographic resolution: partial versus full viral genomes

**Figure 1.**
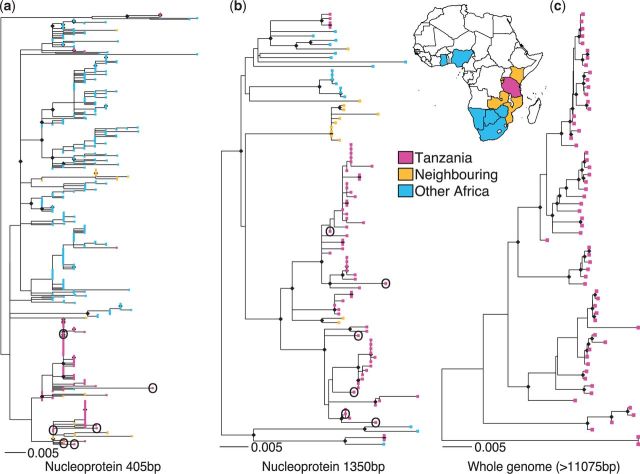
ML trees derived from datasets of rabies virus sequences from the Africa 1b clade for increasing levels of genome coverage: (a) 430 sequences from African countries highlighted on the map for a 405 bp fragment of the nucleoprotein gene, (b) 100 sequences of full 1,350 bp nucleoprotein gene from the same countries (except Botswana, Ghana, Kenya, and Zimbabwe); and (c) sixty full or near-full genome sequences (range: 11,076–11,923 bp) from Tanzania. Trees are scaled by number of substitutions per site and node symbols indicate nodes with bootstrap support ≥ 0.8. Historical samples from the Serengeti District (∼20 years old) are circled in partial genome trees.

**Figure 2.**
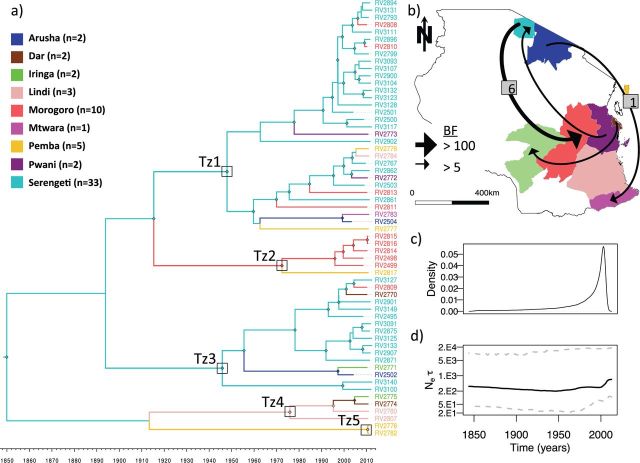
Regional phylogeography among sixty rabies virus whole-genome sequences sampled in Tanzania from 2003 to 2012: (a) an MCC tree with branches coloured according to the most probable posterior location of its descendent node inferred by discrete-state phylogeographic reconstruction in BEAST. Five major phylogenetic groups (Tz1-5) are annotated on the tree and node symbols indicate node posterior support ≥0.9. (b) The four most significant dispersal pathways indicated by BF results from a BSSVS procedure in BEAST with the median number of transitions estimated by Markov jump counts indicated in cases where posterior support for a transition was >0.7. (c) Markov jump densities for total number of transitions through time. (d) Bayesian Skyline plot showing *N*e*τ*, the product of the effective population size (*N*e), and the generation time in years (*τ*) through time.

Consistent with previous large-scale phylogenetic studies, partial genome phylogenies indicated that RABV in sub-Saharan Africa falls into several regional groups with viruses from Eastern Africa generally being genetically distinct from those in western, central, and southern parts of the continent (Supplementary Figs S1 and S2). Of the four major lineages of RABV in Africa (David et al. 2007; Bourhy et al. 2008; Talbi et al. 2009), only the Cosmopolitan clade, and more specifically the Africa 1b lineage, was detected in Tanzania, as found previously (Lembo et al. 2007). Within the Africa 1b lineage, there was evidence of admixture between Tanzania and neighbouring countries and occasional long-range admixture at a continental scale (ML trees in Fig. 1 and Bayesian MCC trees in Supplementary Fig. S3). Sequences clustering most closely with Tanzanian sequences came from Kenya, which shares a border to the north. Although partial genome data were sufficient to identify such large-scale spatial patterns, these data did not provide adequate resolution to distinguish between samples at sub-national scales within Tanzania. The proportion of nodes with bootstrap support ≥80 per cent was only 0.11 (Bayesian posterior probability ≥ 90%: 0.18) for partial N and 0.27 (Bayesian: 0.41) for full N gene phylogenies. Furthermore, out of the sixty Tanzanian WGS, 60 per cent were identical at the partial N and 25 per cent at the full N gene level. In contrast, ML and Bayesian trees based on whole-genome sequences were fully resolved and well supported (proportion of nodes with ML bootstrap support ≥ 80%: 0.86; Bayesian posterior probability ≥90%: 0.89), even when samples had been taken in close spatial and temporal proximity. For example, RV2498 and RV2499 were sampled a day apart from the same village in Morogoro region: both samples only differed by a single SNP for the full N gene but were differentiated by twenty-five SNPs in the whole-genome alignment. This large divergence strongly suggests that the two samples are not from the same chain of transmission, which might have been the conclusion based on partial genome data. The median raw pairwise genetic distance between Tanzanian whole-genome sequences was 259 (range: 0–608) nucleotides and between the Serengeti District samples was 120 (range: 2–212) nucleotides, showing considerable diversity at even a small spatiotemporal scale. However, much of this high divergence is due to the presence of multiple lineages, some of which are evident even based on partial genome data. Using WGS, we identified five distinct lineages with high posterior probability support (annotated in Fig*.* 2)—median pairwise genetic distance within each lineage is listed in Table 1. Bayesian phylogenetic reconstruction of WGS yielded a mean evolutionary rate of 1.44 × 10^−^^4^ substitutions/site/year (95% highest posterior density: 5.78 × 10^−7^ to 3.19 × 10^−4^), similar to previous estimates for N gene and G gene evolution (David et al. 2007; Talbi et al. 2009; Ahmed et al. 2015).

**Table 1.** Raw median genetic distance within each of the five main rabies virus lineages identified in Tanzania.

	Genetic distance	
RV lineage	No. of substitutions per site	No. of SNPs
Tz1	9.47E-03	110
Tz2	2.27E-03	27
Tz3	8.83E-03	95
Tz4	4.17E-03	49.5
Tz5	0	0

### 3.2 Phylogeography of RABV in Tanzania

**Figure 3.**
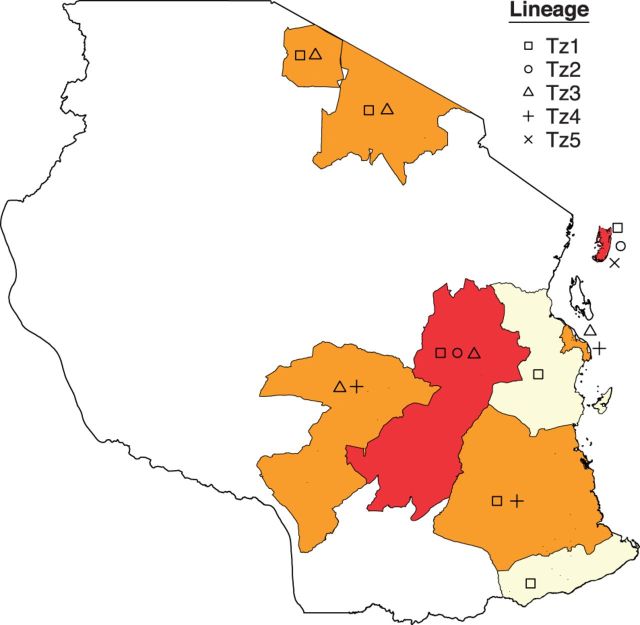
Spatial distribution of rabies virus lineages sampled from regions in Tanzania between 2003 and 2012 with a colour gradient (yellow to red) indicating the total number of lineages (low to high) sampled in each region.

Within Tanzania, we found evidence of phylogeographic structure (AI = 0.70, *P* < 0.001), similar to other estimates of African intra-country AI values (Table 2) (Talbi et al. 2009). Compared with the strong spatial structure between countries and larger spatial aggregations (Table 3), this indicates more fluid and dynamic dispersal patterns within Tanzania, as has been found in other African countries (Talbi et al. 2010). Across the posterior distribution of trees, there were 24 (95% highest posterior density: 21–28) independent lineage movement events. Using a summarized history of Markov Jump counts across the phylogeny, we found that 43 per cent of these migrations occurred in the most recent ten years of the phylogeny (Fig. 2c). A BSSVS procedure in BEAST identified eighteen potential diffusion pathways to explain the observed phylogeographic patterns in the posterior distribution. However, only four received substantial support based on BF > 5 (Fig*.* 2). Support was particularly strong for dispersal from the Serengeti District to Morogoro (BF = 135.30), and Markov jump counts estimated a median of six (range: 3–10) migrations along this dispersal route, with at least one (range: 1–3) occurring on a branch representing a period of less than 2 years. Most lineages were sampled in more than one region in Tanzania, with some distributed across a larger geographic area than others (Fig*.* 3). The largest lineage, Tz1, contains not only a cluster of Serengeti samples but also encompassed samples from a larger geographic extent and was found in eight out of the nine districts sampled. The Bayesian skyline plot revealed that the effective population size has remained fairly constant over the past 150 years (Fig*.* 2d). Because of the much higher availability of samples from the Serengeti District, we also conducted a coarser phylogeographic analysis grouping sequences into ‘North’, ‘South’, or ‘Pemba Island’. This identified a predominance of north to south dispersal (estimated thirteen independent movements compared with one movement south to north) and evidence of dispersal back and forth between the North of mainland Tanzania and Pemba Island (Supplementary Fig. S4).

**Table 2.** Degree of within-country rabies virus spatial admixture in three African countries measured using an Association Index (AI), which ranges from 0 indicating strong population subdivision to 1 indicating complete panmixis. Results are shown for Tanzania (this study) and Algeria and Morocco (Talbi et al*.* 2010).

Country	AI [95% BCI]	*P*	No of sequences	No of locations	Median distance (km)	Min	Max
Algeria	0.67 [0.62–0.73]	<0.001	117	20	233.08	23.27	674.62
Morocco	0.55 [0.51–0.63]	<0.001	133	28	326.45	28.62	926.70
Tanzania	0.70 [0.60-0.79]	<0.001	60	9	439.76	39.05	1088.26

BCI, Bayesian confidence interval.

**Table 3.** Degree of spatial admixture between rabies virus samples from Africa according to an Association Index (AI). AI values can range from 0 indicating complete population subdivision to 1 indicating complete panmixis.

Data	Spatial clustering level	No. of groups	AI [95% BCI]	*P*
N405	Sub-continent	3	0.04 [0.02−0.05]	<0.01
	Country	14	0.06 [0.05−0.08]	<0.01
N1350	Sub-continent	3	0.06 [0.04−0.08]	<0.01
	Country	10	0.13 [0.09−0.13]	<0.01

BCI, Bayesian confidence interval. Datasets of partial (N405) and full (N1350) nucleoprotein sequences were tested at two levels of spatial aggregation: (1) sub-continent geographical partitions relative to Tanzania (three states: Tanzania, neighbouring country, and other African country) and (2) country of origin.

## 4 Discussion

Using viral genetic data from a hierarchy of spatial scales and varying levels of genome coverage, we were able to demonstrate the advantages of whole-genome resolution to describe the spatio-temporal dynamics of endemically circulating canine rabies viruses.

We found a clear phylogeographic structure between countries in Africa, which could be identified with partial genome data. This suggests that the majority of dispersal occurs at a within-country scale, and control programs would be most appropriate at a national scale with strategies to deal with potential incursions from other countries. However, at regional and local scales within Tanzania, the discriminatory power of partial genome data became too limited to reveal fine-scale population structure that could aid the effectiveness of control interventions. In contrast, WGS provided the resolution to genetically distinguish between all samples and produced a well-supported phylogeny. Although sub-genomic information has utility at broad phylogeographic scales (e.g., Horton et al. 2015), this finding supports the application of WGS for studies aiming to discern population structure at a scale most relevant to control.

Although large-scale population structure according to sub-continental areas or country-specific lineages was apparent from sub-genomic data, we also found incidences of occasional large-scale admixture (Supplementary Figs S1 and S2). We continued to find evidence at increasingly fine scales: Tanzania and other countries within the Africa 1b clade showed signs of admixture particularly when countries shared a border and within country movements of RABV facilitated by humans were a feature of Tanzanian RABV. Even at a very local scale, within the Serengeti District, we found multiple co-circulating lineages. This recurrent theme may be a characteristic of endemic RABV in Africa reflecting decades of endemic circulation and human-mediated introductions. Similar patterns have been observed in canine rabies-endemic countries in South and Southeast Asia (Lumlertdacha et al. 2006; Matsumoto et al. 2013; Ahmed et al. 2015), where the presence of co-circulating lineages was attributed to a combination of historical introductions from neighbouring countries and human-mediated movements.

Much of the viral population structure we found within Tanzania is consistent with initial invasive waves that have persisted endemically, with structure eroding over time aided by human-mediated movement. Historical accounts describe a rabies outbreak in southern regions of Tanzania in the mid-1950s, which spread throughout the country and was recorded in the Serengeti District in the late 1970s (Magembe 1985; Siongok and Karama 1985). This invasion perhaps shaped the initial RABV population structure in Tanzania. The timeline of regional scale migrations (Fig*.* 2c) indicates a strong rise in viral dispersal around this period, possibly reflecting increasing human connectivity. Samples in Tz1 (Fig*.* 2) share partial N gene identity with samples from Kenya (Fig*.* 1), indicating a shared evolutionary history that may relate to an initial outbreak across both countries. Lineages Tz1 and Tz3 appear to have a wide geographic distribution (Fig*.* 3), and we found evidence of a general north to south dispersal pattern in Tanzania (Supplementary Fig. S4). This highlights the potential for widespread dispersal should an incursion occur from Kenya and suggests a need for co-operative, cross-border rabies management once a national control program is established. Variation in the spread of different lineages across Tanzania may reflect the influence of heterogeneous landscape features or dog population structure in impeding or facilitating RABV dissemination. Identifying and quantifying such features is the logical next step to provide additional information that can inform control programmes, such as identifying and strengthening pre-existing barriers. Recent extensions of phylogeographic techniques have highlighted how this might be achieved using an integrated approach combining evolutionary and ecological analyses to quantify drivers of viral transmission (Lemey et al. 2014; Magee et al. 2014).

The ancestry of lineages Tz4 and 5 (denoting lineages found in the southern mainland and on the Island of Pemba, respectively) points toward an introduction from the north of Tanzania (posterior probability = 0.62), but the uncertainty of this ancestral location and the ancestral node itself (posterior = 0.45) suggest these may be distinct historical lineages with low-level persistence or undetected circulation elsewhere in Tanzania. Alternatively, these clusters may represent instances where lineages from external sources have more recently invaded and resulted in sustained transmission in Tanzania—partial genome phylogenies indicate Tz4 is related to clusters containing samples from countries south of Tanzania, South Africa and Mozambique. Furthermore, Tz5 shares a common ancestry with a Nigerian and several Central African Republic samples. This again underlines the threat of re-invasion or introduction from external sources and the potential value of whole-genome resolution to robustly and more accurately identify sources of new introductions. To date, the majority of RABV genetic data from Africa comes from partial genome analyses; however, our data suggest that whole-genome characterization would be valuable and should be an aim for the future.

Islands represent isolated landscapes with natural barriers to dispersal, but incidents of RABV outbreaks instigated by human-mediated introductions (e.g., to islands in Indonesia [Windiyaningsih et al. 2004; Susilawathi et al. 2012]) have often been recorded. Sequences from the island of Pemba were suggestive of multiple introductions from various sources with evidence of dispersal to and from the mainland (Supplementary Fig. S4). Samples from Pemba were scattered throughout the phylogeny, and the most divergent lineage Tz5 consisted of two samples from Pemba. The distribution of these lineages may reflect earlier invasions of RABV into Pemba from elsewhere in Tanzania (Tz1 and 2) and the African continent (Tz5), resulting from Pemba’s location on an important trading route. However, since these lineages were not resampled and no cases have been detected from Pemba in over a year (Lushasi pers. comm.), RABV may have been only transiently circulating. Nevertheless, lessons from Indonesia, where lack of a swift and coordinated response to a rabies incursion led to an epidemic (Townsend et al. 2013), highlight the importance of active surveillance and rapid response to incursions.

Although we found evidence of RABV spatial structure within Tanzania, it was evident that dispersal was also frequent, with at least one long distance migration occurring within a small temporal window (<2 years). Lineage movements occurring on branches representing short evolutionary times, such as those identified between Serengeti and Morogoro (>750 km), indicate rates of dispersal much higher than those recorded for endemic wildlife rabies (Biek 2007) and imply human mediated movements as seen in parts of North Africa (Talbi et al. 2010). Movement of pastoralist and agro-pastoralist communities from the Lake zone in Northern Tanzania to southern regions, for example, Morogoro have been ongoing since the 1950s (Walsh 2008), with a major influx reported from 2003 to 2006 (PINGO’s Forum 2013), attributed to climate change induced drought and forced evictions from newly protected areas (Kideghesho et al. 2013). During these migrations, pastoralists are accompanied by their dogs, which may facilitate the long distance movement of animals incubating the virus prior to transmission. On further inspection, we found that Morogoro samples indicated as instances of long-distance translocation came from rural areas where pastoralists are likely to migrate to, whereas other samples from urban Morogoro formed a distinct cluster (Fig*.* 2). We found three RABV lineages in Morogoro from only ten samples (Fig*.* 3). Quantifying networks of human-mediated movements (including livestock trade) would provide a valuable proxy for connectivity for many zoonotic diseases affecting domesticated animals.

Further to our findings of regional admixture in Tanzania, we also observed considerable diversity at a very local scale, that is, within the Serengeti District, with several lineages co-circulating. These lineages appear to have persisted for at least 20 years (older Serengeti samples obtained from GenBank also cluster within these lineages, indicated in Fig*.* 1) despite dog vaccination campaigns having been conducted in the district since 1996. While vaccination coverage has varied substantially across years and between villages (Viana et al. 2015) rabies incidence has at times significantly declined, for example, falling by 97 per cent in the late 1990s (Cleaveland et al. 2003). Yet our genetic data show that, despite substantial declines in incidence, these lineages must have persisted at very low levels within the district or subsequently reinvaded from neighbouring districts. Without sampling the surrounding districts, it is not possible to distinguish between these possibilities. The skyline plot indicated a stable effective RABV population size (*N*_e_) through time (Fig*.* 3d). While this could be expected for an endemic pathogen, it is worth noting that there was no evidence of viral population size reductions in response to vaccination campaigns. Rabies control efforts across much of Tanzania have been very limited until recently and high turnover in the dog population (Hampson et al. 2009) likely contributes to the stable persistence of rabies in Tanzania. It has also been noted that geographic structure can obscure local fluctuations in subpopulations while maintaining the appearance of a constant *N*_e_ in skyline plots (Carrington et al. 2005).

This study represents a snapshot of RABV dynamics in Tanzania, indicating that human movements have disseminated RABV out of locally endemic areas at scales relevant to control, that is, administrative units such as regions or districts. These frequent translocations have probably lead to the existence of multiple co-circulating lineages (Fig. 3), but relatively few introductions lead to sustained chains of transmission that are detectable as lineage movement events. However, in disease systems closely associated with human activities, the probability of successful translocation and establishment is likely to be much greater. This suggests that without some level of regional co-operation, Tanzania will be unable to eliminate rabies and maintain freedom from disease. Human movements are often in response to social drivers, which could be used as signals for increased vigilance and surveillance in at risk areas.

Our findings highlight the use of WGS to uncover fine scale transmission patterns that can directly inform control efforts. However, sub-genomic approaches can still have utility at a coarser scale and are more easily obtained, particularly when sample quality is an issue. In particular, they can be used to initially identify admixture between countries, which may indicate the necessity of coordinated regional control programs and surveillance. Co-circulation of multiple lineages and introductions facilitated by humans appear to be a feature of endemic rabies virus and complicate the design of a sustainable control strategy. However, using whole-genome data, we were able to identify sources of dispersal within Tanzania that could direct efforts toward surveillance and control. The finding that humans play an important role in the dynamics of RABV in Tanzania suggests that increasing awareness and dog vaccination in ‘high-risk’ communities such as pastoralists could help to reduce long-range dispersal. Moreover, the design of enhanced surveillance and containment strategies to mitigate human-mediated incursions and maintain disease freedom should be a priority once control programs are established and elimination is being targeted.

## Supplementary data

Supplementary data is available at *VEVOLU Journal *online.

Supplementary Table S1
