# Supplementary material for: Elucidating the phylodynamics of endemic rabies virus in eastern Africa using whole-genome sequencing
Source: Virus Evol. 2015 Sep 10;1(1):vev011. doi: 10.1093/ve/vev011 (PMC5014479; doi:10.1093/ve/vev011)
Supplement: Supplementary Table S1 [file S1_Table.docx]

Table S1. Epidemiological information and whole genome sequencing (WGS) details for Tanzanian whole genome samples used in this study (*reference sequence).

| **Region** | **Date** | **Easting** | **Northing** | **Species** | **WGS protocol** | **Sample ID** | **% Genome coverage** | **Average depth of coverage** | **Accession no** |
| --- | --- | --- | --- | --- | --- | --- | --- | --- | --- |
| Arusha | 18-Apr-04 | 777032 | 9793554 | Cow | Miseq | RV2502 | 100 | 165 | KR906739 |
| Arusha | 18-Jun-03 | 784690 | 9771564 | Domestic dog | Miseq | RV2504 | 100 | 1871 | KR906741 |
| Dar es Salaam | 03-May-10 | 1207114 | 9234522 | Domestic dog | Miseq | RV2770 | 100 | 22 | KR906743 |
| Dar es Salaam | 02-Nov-10 | 1182523 | 9234308 | Domestic dog | Miseq | RV2774 | 100 | 146 | KR906746 |
| Iringa | 27-May-10 | 750553 | 9053269 | Domestic dog | Miseq | RV2771 | 100 | 42 | KR906744 |
| Iringa | 26-Nov-10 | 750553 | 9053269 | Domestic dog | Miseq | RV2775 | 100 | 120 | KR906747 |
| Lindi | 07-Feb-11 | 1088355 | 8845396 | NA | Depleted RNA: 454 | RV2780 | 100 | 20 | KR906751 |
| Lindi | 28-Feb-11 | 1088355 | 8845396 | NA | Miseq | RV2784 | 100 | 27 | KR906754 |
| Lindi | 03-Oct-10 | 1088355 | 8845396 | NA | Depleted RNA: 454 | RV2807 | 100 | 10 | KR906757 |
| Morogoro | 06-Aug-08 | 244890 | 9075488 | Domestic dog | Miseq | RV2498 | 100 | 61 | KR906735 |
| Morogoro | 05-Aug-08 | 243727 | 9075748 | Domestic dog | Miseq | RV2499 | 100 | 528 | KR906736 |
| Morogoro | 04-Apr-10 | 1040336 | 9253119 | Domestic dog | Miseq/Nextseq | RV2808 | 99 | 28 | KR906758 |
| Morogoro | 16-Apr-10 | 1032182 | 9228490 | Domestic dog | Miseq | RV2809 | 98 | 20 | KR906759 |
| Morogoro | 23-Apr-10 | 1065994 | 9258751 | Domestic dog | Miseq | RV2810 | 100 | 101 | KR906760 |
| Morogoro | 24-Apr-10 | 1033809 | 9250464 | Domestic dog | Miseq/Nextseq | RV2811 | 98 | 16 | KR906761 |
| Morogoro | 28-Apr-10 | 352510 | 9245700 | NA | Miseq | RV2813 | 100 | 36 | KR906762 |
| Morogoro | 17-May-10 | 898066 | 8996235 | Pig | Miseq | RV2814 | 100 | 962 | KR906763 |
| Morogoro | 27-Sep-10 | 9105076 | 242926 | Cow | Miseq | RV2815 | 100 | 77 | KR906764 |
| Morogoro | 28-Sep-10 | 242926 | 9105076 | Cow | Miseq | RV2816 | 100 | 117 | KR906765 |
| Mtwara | 28-Feb-11 | 1193634 | 8807598 | NA | Miseq | RV2783 | 100 | 153 | KR906753 |
| Pemba | 29-Dec-12 | 1256936 | 9414352 | Domestic dog | Miseq | RV2776 | 100 | 499 | KR906748 |
| Pemba | 24-Dec-12 | 1256936 | 9414352 | Domestic dog | Miseq/Nextseq | RV2777 | 100 | 57 | KR906749 |
| Pemba | 26-Dec-12 | 1256936 | 9414352 | Domestic dog | Miseq | RV2778 | 99 | 41 | KR906750 |
| Pemba | 11-Feb-11 | 1205189 | 9313425 | Domestic dog | Miseq | RV2782 | 100 | 226 | KR906752 |
| Pemba | 26-Nov-10 | 1256936 | 9414352 | Domestic dog | Miseq | RV2817 | 100 | 44 | KR906766 |
| Pwani | 02-Aug-10 | 1138047 | 9246282 | Domestic dog | Genbank | RV2772 | na | na | KF155002* |
| Pwani | 26-Oct-10 | 1138047 | 9246282 | Domestic dog | Miseq/Nextseq | RV2773 | 100 | 104 | KR906745 |
| Serengeti | 12-Jul-08 | 696385 | 9802796 | Domestic dog | Miseq | RV2495 | 100 | 129 | KR906734 |
| Serengeti | 23-Nov-08 | 698019 | 9804712 | Domestic dog | Miseq | RV2500 | 100 | 16 | KR906737 |
| Serengeti | 14-Feb-04 | 679372 | 9810508 | Domestic dog | Miseq | RV2501 | 100 | 108 | KR906738 |
| Serengeti | 15-Feb-09 | 686729 | 9761151 | Wild Cat | Miseq | RV2503 | 100 | 29 | KR906740 |
| Serengeti | 12-Sep-09 | 665178 | 9777064 | Domestic dog | Miseq | RV2767 | 100 | 250 | KR906742 |
| Serengeti | 02-Jan-11 | 657176 | 9822908 | Cow | Miseq | RV2793 | 100 | 39 | KR906755 |
| Serengeti | 29-Jan-11 | 680129 | 9805797 | Domestic dog | Miseq | RV2799 | 100 | 41 | KR906756 |
| Serengeti | 11-May-11 | 691423 | 9791388 | Domestic dog | Miseq | RV2861 | 100 | 1259 | KR906767 |
| Serengeti | 11-May-11 | 674259 | 9805687 | Domestic dog | Miseq | RV2862 | 100 | 21 | KR906768 |
| Serengeti | 17-Jun-11 | 701315 | 9794641 | Domestic dog | Amplicon seq: 454 | RV2871 | 100 | 25 | KR906769 |
| Serengeti | 29-Jun-11 | 680196 | 9811835 | Domestic dog | Miseq | RV2875 | 100 | 99 | KR906770 |
| Serengeti | 15-Aug-11 | 653024 | 9822353 | Domestic dog | Miseq | RV2894 | 100 | 1470 | KR906771 |
| Serengeti | 19-Aug-11 | 681869 | 9798035 | Domestic dog | Miseq | RV2896 | 100 | 69 | KR906772 |
| Serengeti | 27-Sep-11 | 656396 | 9803751 | Domestic dog | Miseq | RV2900 | 100 | 84 | KR906773 |
| Serengeti | 22-Sep-11 | 653179 | 9802910 | Domestic dog | Miseq | RV2901 | 100 | 107 | KR906774 |
| Serengeti | 24-Sep-11 | 684532 | 9790009 | Domestic dog | Miseq | RV2902 | 99 | 44 | KR906775 |
| Serengeti | 16-Oct-11 | 700560 | 9803728 | Cow | Miseq | RV2907 | 100 | 71 | KR906776 |
| Serengeti | 05-Dec-11 | 696698 | 9792023 | Domestic dog | Miseq/Nextseq | RV3091 | 100 | 497 | KR906777 |
| Serengeti | 22-Dec-11 | 681720 | 9798404 | Domestic dog | Miseq | RV3093 | 100 | 43 | KR906778 |
| Serengeti | 19-Feb-12 | 698140 | 9804256 | Domestic dog | Miseq | RV3100 | 100 | 24 | KR906779 |
| Serengeti | 22-Dec-11 | 648913 | 9823033 | Sheep | Miseq | RV3104 | 100 | 95 | KR906780 |
| Serengeti | 09-Apr-12 | 656704 | 9803322 | Domestic dog | Miseq | RV3107 | 100 | 293 | KR906781 |
| Serengeti | 29-Apr-12 | 650984 | 9807685 | Domestic dog | Miseq | RV3111 | 100 | 27 | KR906782 |
| Serengeti | 12-May-12 | 669765 | 9795977 | Domestic dog | Miseq | RV3117 | 100 | 58 | KR906783 |
| Serengeti | 26-Apr-12 | 647442 | 9799177 | Domestic dog | Miseq | RV3123 | 100 | 65 | KR906792 |
| Serengeti | 06-Jun-12 | 658003 | 9809433 | Goat | Miseq/Nextseq | RV3125 | 100 | 106 | KR906784 |
| Serengeti | 11-Jun-12 | 681576 | 9797817 | Domestic dog | Miseq | RV3127 | 100 | 204 | KR906785 |
| Serengeti | 16-Jun-12 | 674458 | 9797076 | donkey | Miseq | RV3128 | 100 | 89 | KR906786 |
| Serengeti | 07-Jul-12 | 685498 | 9797037 | Domestic dog | Miseq | RV3131 | 100 | 113 | KR906787 |
| Serengeti | 02-Apr-12 | 656028 | 9801566 | Domestic dog | Miseq | RV3132 | 100 | 14 | KR906788 |
| Serengeti | 04-Jul-12 | 700856 | 9800221 | Domestic dog | Miseq/Nextseq | RV3133 | 100 | 118 | KR906789 |
| Serengeti | 27-Jul-12 | 656945 | 9804374 | Domestic dog | Miseq | RV3140 | 100 | 738 | KR906790 |
| Serengeti | 23-Jan-12 | 657742 | 9809576 | Domestic dog | Miseq | RV3149 | 93 | 6 | KR906791 |
