# Supplementary material for: Elucidating the phylodynamics of endemic rabies virus in eastern Africa using whole-genome sequencing
Source: Virus Evol. 2015 Sep 10;1(1):vev011. doi: 10.1093/ve/vev011 (PMC5014479; doi:10.1093/ve/vev011)
Supplement: Supplementary Table S1 [file S2_Table.docx]

Table S2. Relevant epidemiological data for Rabies virus partial nucleoprotein sequences (405bp) from Tanzania generated for this study, including GenBank accession numbers.

| **Sample ID** | **Region** | **Species** | **Year** | **Accession.no** |
| --- | --- | --- | --- | --- |
| RV2490 | Serengeti | Domestic dog | 2008 | KR534217 |
| RV2491 | Serengeti | Domestic dog | 2008 | KR534218 |
| RV2492 | Serengeti | Domestic dog | 2007 | KR534219 |
| RV2493 | Serengeti | Domestic dog | 2008 | KR534220 |
| RV2494 | Serengeti | Cat | 2009 | KR534221 |
| RV2496 | Serengeti | Honey badger | 2004 | KR534222 |
| RV2497 | Serengeti | Domestic dog | 2007 | KR534223 |
| RV2768 | na | Domestic dog | na | KR534224 |
| RV2769 | Iringa | Domestic dog | 2010 | KR534225 |
| RV2779 | Pwani | Domestic dog | 2011 | KR534226 |
| RV2787 | Serengeti | Domestic dog | 2010 | KR534227 |
| RV2788 | Serengeti | Domestic dog | 2010 | KR534228 |
| RV2789 | Serengeti | Domestic dog | 2010 | KR534229 |
| RV2790 | Serengeti | Domestic dog | 2010 | KR534230 |
| RV2791 | Serengeti | Domestic dog | 2010 | KR534231 |
| RV2792 | Serengeti | Jackal | 2011 | KR534232 |
| RV2794 | Serengeti | Domestic dog | 2011 | KR534233 |
| RV2795 | Serengeti | Cow | 2011 | KR534234 |
| RV2796 | Serengeti | Cow | 2011 | KR534235 |
| RV2797 | Serengeti | Cow | 2011 | KR534236 |
| RV2798 | Serengeti | Cow | 2011 | KR534237 |
| RV2800 | Serengeti | Domestic dog | 2011 | KR534238 |
| RV2802 | na | Na | 2011 | KR534239 |
| RV2804 | na | Na | na | KR534240 |
| RV2806 | na | Na | na | KR534241 |
| RV2856 | na | Na | na | KR534242 |
| RV2857 | Serengeti | Domestic dog | 2011 | KR534243 |
| RV2889 | Serengeti | Domestic dog | 2011 | KR534244 |
| RV2890 | Serengeti | Cow | 2011 | KR534245 |
| RV2891 | Serengeti | Civet | 2011 | KR534246 |
| RV2892 | Serengeti | Domestic dog | 2011 | KR534247 |
| RV2893 | Serengeti | Cow | 2011 | KR534248 |
| RV2895 | Serengeti | Domestic dog | 2011 | KR534249 |
| RV2897 | Serengeti | Domestic dog | 2011 | KR534250 |
| RV2898 | Serengeti | Domestic dog | 2011 | KR534251 |
| RV2899 | Serengeti | Domestic dog | 2011 | KR534252 |
| RV2903 | Serengeti | Domestic dog | 2011 | KR534253 |
| RV2906 | Serengeti | Cat | 2011 | KR534254 |
| RV2908 | Serengeti | Goat | 2011 | KR534255 |
| RV2909 | Serengeti | Cow | 2011 | KR534256 |
| RV2910 | Morogoro | Na | 2011 | KR534257 |
| RV2911 | Morogoro | Na | 2011 | KR534258 |
| RV2913 | Morogoro | Na | 2011 | KR534259 |
| RV2914 | Morogoro | Na | 2011 | KR534260 |
| RV2915 | Morogoro | Na | 2011 | KR534261 |
| RV2916 | Morogoro | Na | 2011 | KR534262 |
| RV2917 | Morogoro | Na | 2011 | KR534263 |
| RV2920 | Morogoro | Na | 2011 | KR534264 |
| RV2921 | Morogoro | Na | 2011 | KR534265 |
| RV2922 | na | Na | 2011 | KR534266 |
