# Supplementary material for: Elucidating the phylodynamics of endemic rabies virus in eastern Africa using whole-genome sequencing
Source: Virus Evol. 2015 Sep 10;1(1):vev011. doi: 10.1093/ve/vev011 (PMC5014479; doi:10.1093/ve/vev011)
Supplement: Supplementary Table S1 [file S3_Table.docx]

Table S3. Model comparison between gene-specific or gene-linked HKY nucleotide models with different codon position partitioned models (alignment has 5 genes and 1 concatenated non-coding sequence partition). The best HKY model was then compared against a GTR nucleotide model to identify the best model (in bold). Marginal likelihood estimation using path sampling (PS) and stepping stone (SS) sampling in BEAST v1.8.1 was used for model selection.

| **Model description** | **Codon partition model** | **Number of partitions** | **Model** | **PS** | **SS** |
| --- | --- | --- | --- | --- | --- |
| Gene partitioned with gene-specific rate variation | NA | 6 | 5 genes * (HKY + Γ) + Non-coding (HKY + Γ) | -31792.27 | -31794.86 |
| Gene linked & codon position partitioned: among codon position rate heterogeneity, homogeneous rates among genes | CP112 | 2 | Gene linked (HKY112 + CP112 + Γ112) + Non-coding (HKY + Γ) | -30955.51 | -30958.76 |
|  | CP123 | 2 | Gene linked (HKY112 + CP112 + Γ112) + Non-coding (HKY + Γ) | -30888.21 | -30891.53 |
| Gene partioned & codon position partitioned: among codon position rate heterogeneity, heterogeneous rates among genes | CP112 | 6 | 5 genes * (HKY112 + CP112 + Γ112) + Non-coding (HKY + Γ) | -31032.33 | -31037.16 |
|  | CP123 | 6 | 5 genes * (HKY123 + CP123 + Γ123) + Non-coding (HKY + Γ) | -30969.91 | -30975.99 |
|  |  |  |  |  |  |
| Gene linked & codon position partitioned: among codon position rate heterogeneity, homogeneous rates among genes | CP112 | 2 | Gene linked (GTR112 + CP112 + Γ112) + Non-coding (GTR + Γ) | -30920.62 | -30924.50 |
|  | **CP123** | **2** | **Gene linked (GTR112 + CP112 + Γ112) + Non-coding (GTR + Γ)** | **-30831.04** | **-30835.69** |
