# Supplementary material for: Elucidating the phylodynamics of endemic rabies virus in eastern Africa using whole-genome sequencing
Source: Virus Evol. 2015 Sep 10;1(1):vev011. doi: 10.1093/ve/vev011 (PMC5014479; doi:10.1093/ve/vev011)
Supplement: Supplementary Table S1 [file S4_Table.docx]

Table S4. Bayes Factor (BF) support for significant rabies virus diffusion pathways in Tanzania identified under a BSSVS procedure and median (with range) number of transitions along those pathways (shown with posterior probability of transition occurring in the phylogeny) estimated via Markov jump counts in BEAST.

| **From** | **To** | **BF** | **Transitions** | **Posterior probability of transition** |
| --- | --- | --- | --- | --- |
| Serengeti | Morogoro | 135.30462 | 6 (3-10) | 1 |
| Arusha | Mtwara | 7.390997216 | 1 (1-3) | 0.72 |
| Dar | Iringa | 5.897122976 | 1 (1-2) | 0.39 |
| Pwani | Serengeti | 5.218889036 | 1 (1-5) | 0.05 |
| Iringa | Arusha | 4.585531317 | 1 (1-2) | 0.27 |
| Mtwara | Arusha | 4.564096186 | 1 (1-3) | 0.2 |
| Arusha | Iringa | 4.312786018 | 1 (1-3) | 0.61 |
| Pemba | Lindi | 4.238109851 | 1 (1-3) | 0.19 |
| Morogoro | Dar | 4.231368777 | 1 (1-2) | 0.05 |
| Pwani | Pemba | 4.045769968 | 1 (1-2) | 0.07 |
| Iringa | Dar | 4.000301452 | 1 (1-3) | 0.37 |
| Pwani | Lindi | 3.45247866 | 1 (1-2) | 0.04 |
| Pemba | Serengeti | 3.365345019 | 1 (1-6) | 0.11 |
| Lindi | Pemba | 3.240077596 | 1 (1-4) | 0.18 |
| Dar | Lindi | 3.212017693 | 1 (1-4) | 0.08 |
| Morogoro | Pemba | 3.156344464 | 1 (1-4) | 0.53 |
| Pemba | Pwani | 3.068485607 | 1 (1-2) | 0.04 |
| Morogoro | Pwani | 3.068485607 | 1 (1-2) | 0.06 |
