# Supplementary figures and images for: Elucidating the phylodynamics of endemic rabies virus in eastern Africa using whole-genome sequencing
Source: Virus Evol. 2015 Sep 10;1(1):vev011. doi: 10.1093/ve/vev011 (PMC5014479; doi:10.1093/ve/vev011)

RABV clade

- Africa1
- Africa2
- Africa3
- Africa4

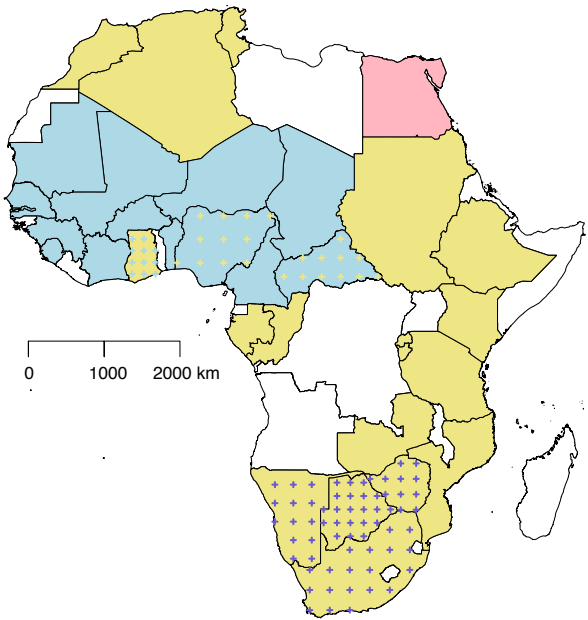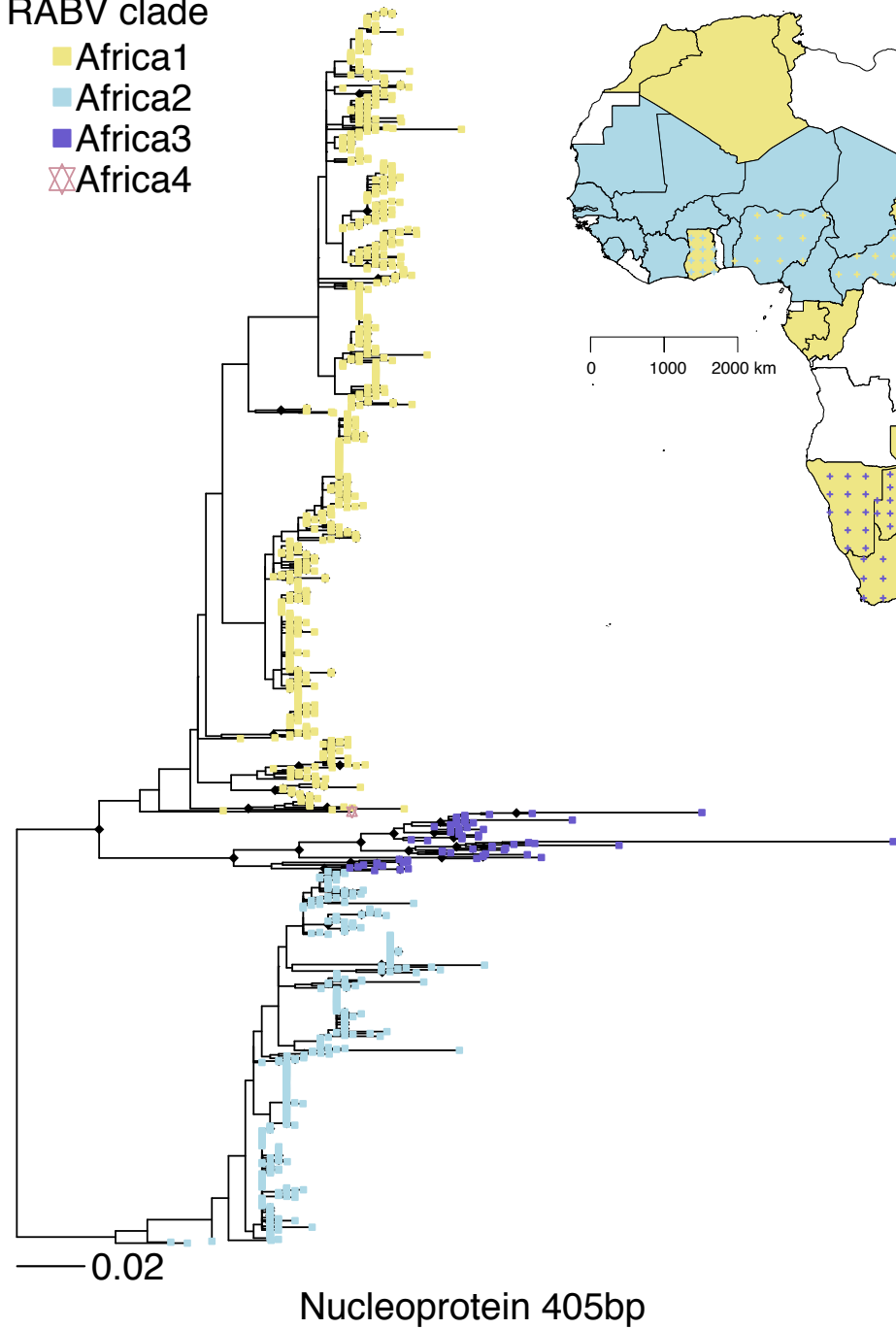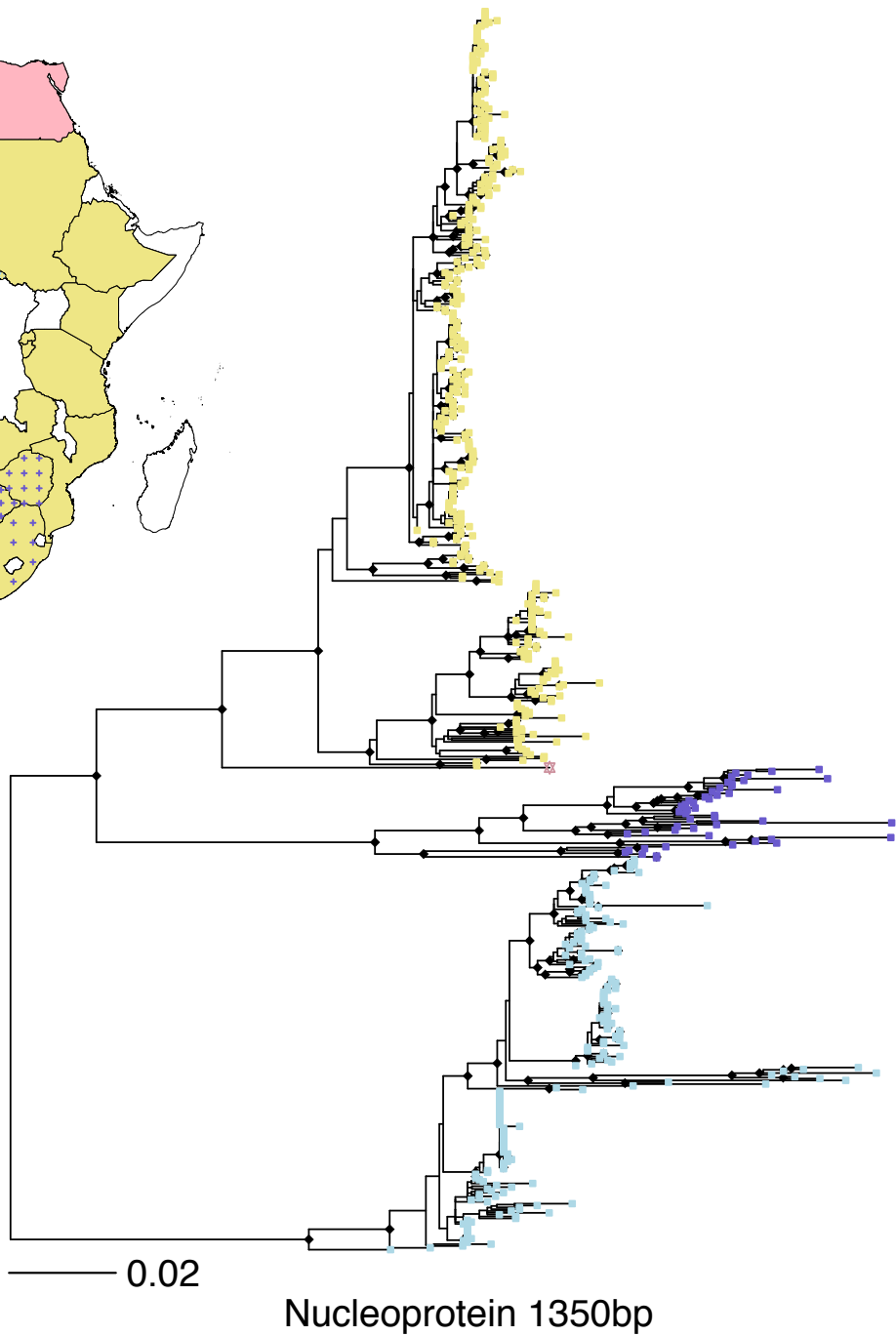

Supplement: Supplementary Table S1 [file S1_Figure.pdf]

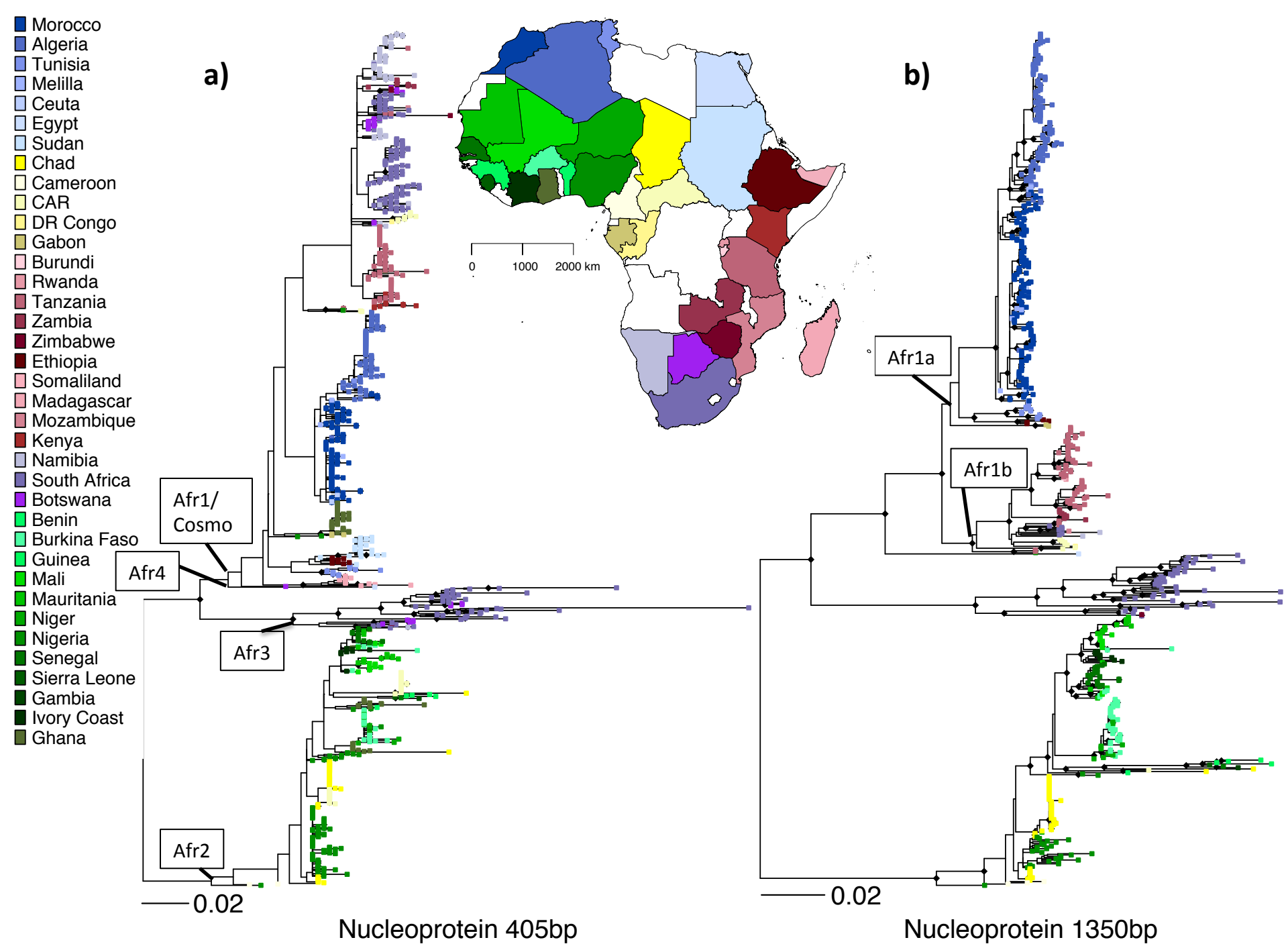

Supplement: Supplementary Table S1 [file S2_Figure.pdf]

a)

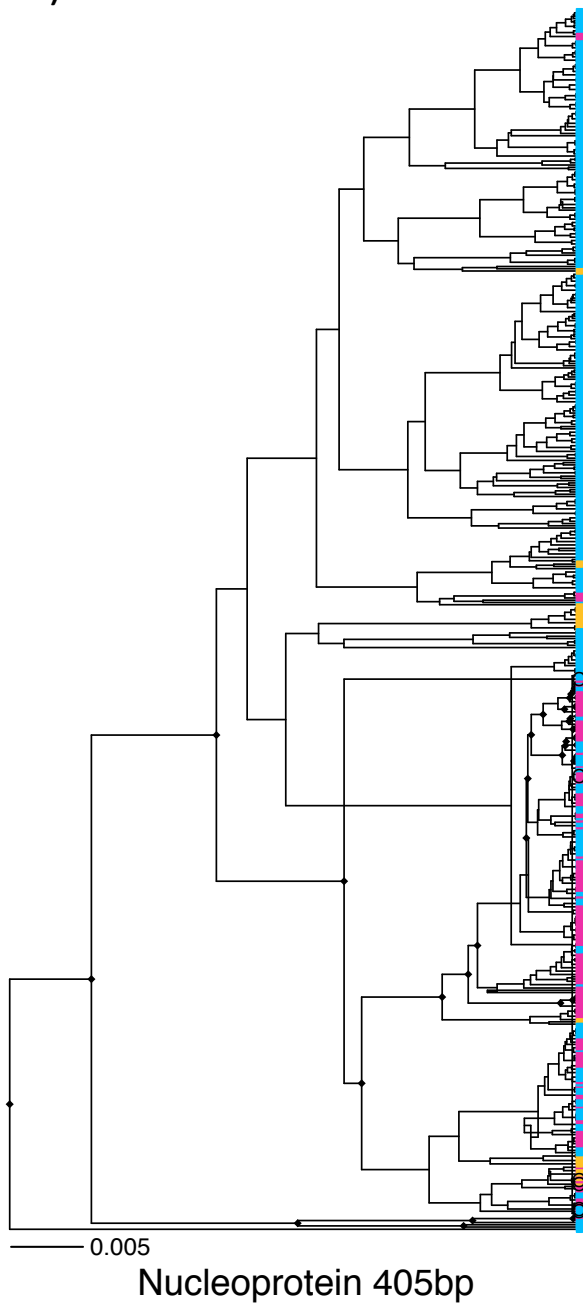

b)

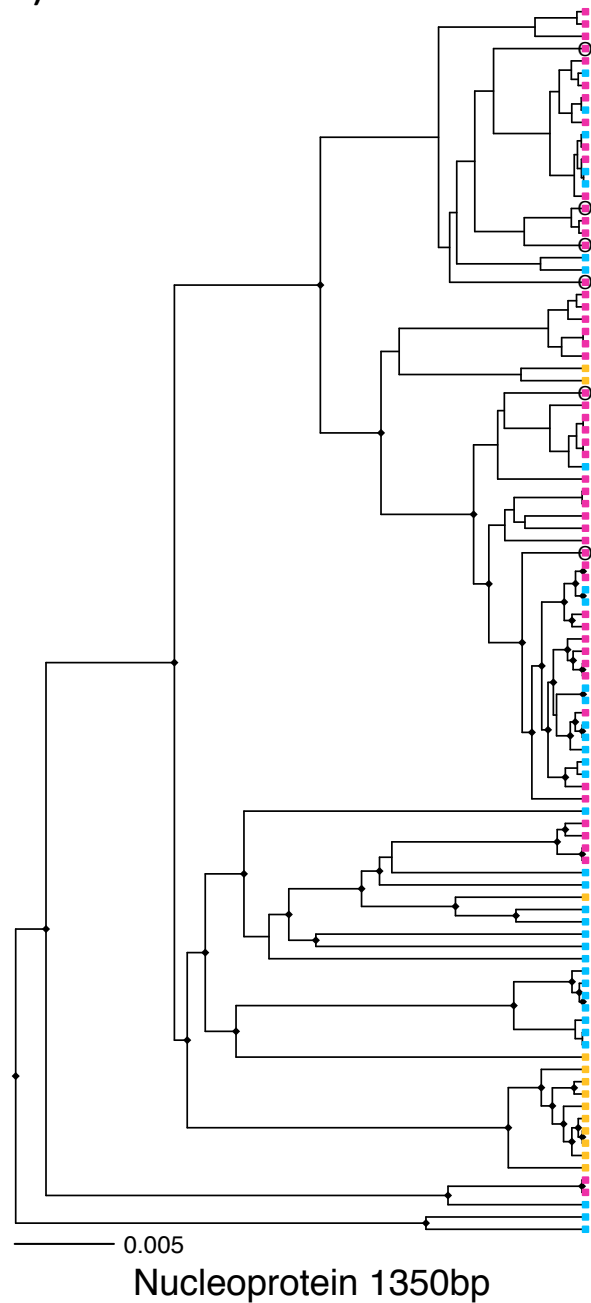

c)

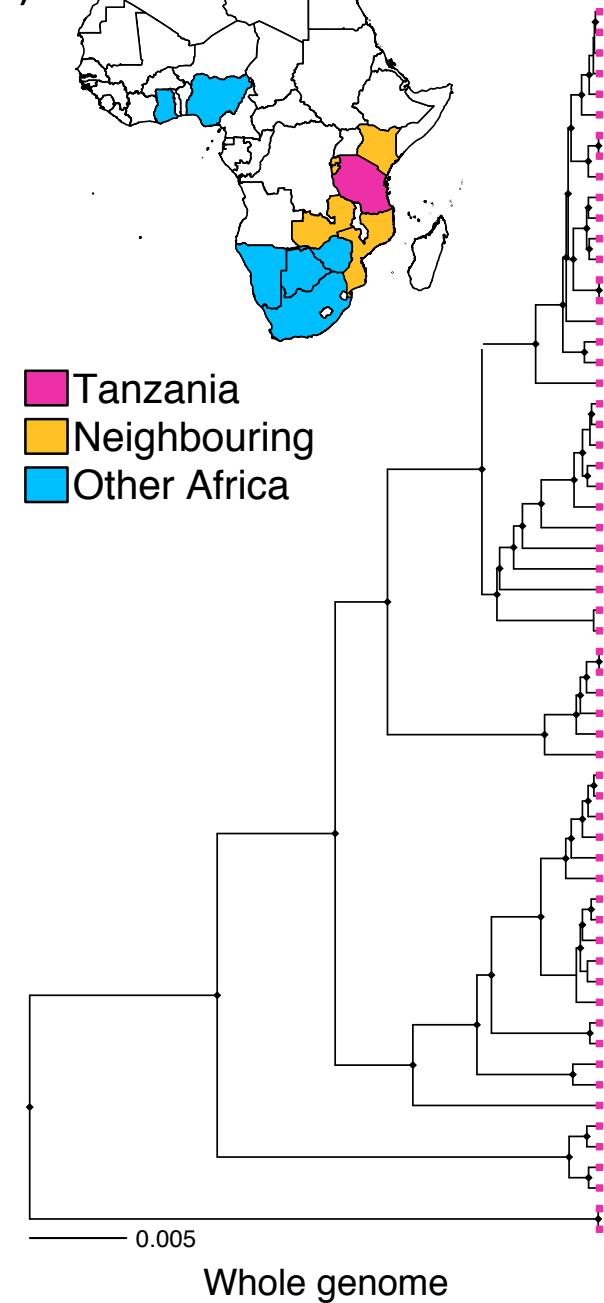

Supplement: Supplementary Table S1 [file S3_Figure.pdf]

| From         | To           | BF     | Transitions |
|--------------|--------------|--------|-------------|
| North        | South        | 7.48   | 13          |
| North        | Pemba island | 9.40   | 3           |
| Pemba island | North        | 219.91 | 1           |

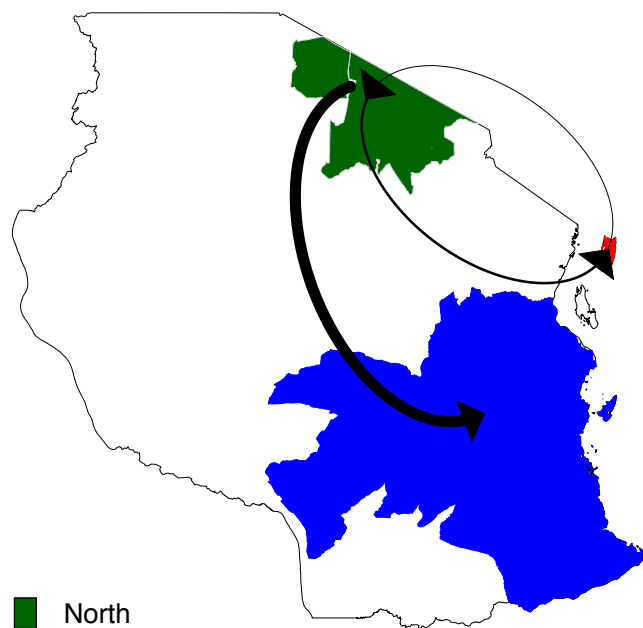

■ North  
■ South  
■ Island

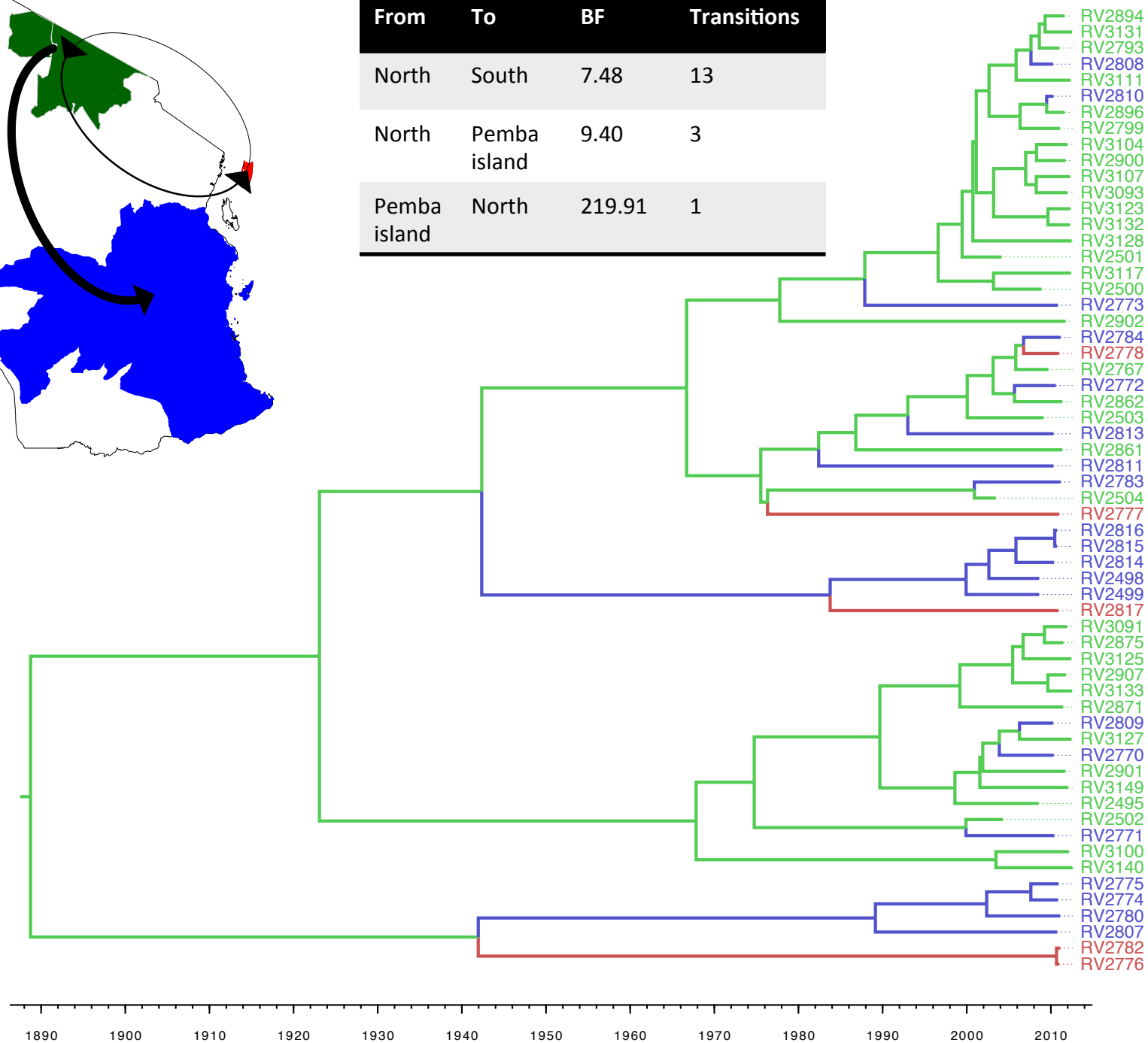

Supplement: Supplementary Table S1 [file S4_Figure.pdf]
