# Supplementary material for: Elucidating the phylodynamics of endemic rabies virus in eastern Africa using whole-genome sequencing
Source: Virus Evol. 2015 Sep 10;1(1):vev011. doi: 10.1093/ve/vev011 (PMC5014479; doi:10.1093/ve/vev011)
Supplement: Supplementary Table S1 [file SI_Figure_legends.docx]

*Figure S1*. Maximum likelihood trees derived from datasets of rabies virus (RABV) sequences from Africa for a) a 405bp fragment of the nucleoprotein (N) gene (n=1317) and b) full length 1350bp nucleoprotein gene sequences (n=674). Samples are colour-coded according to major RABV clades in Africa and their spatial distribution indicated on the map. Countries in which more than one clade was sampled have coloured crosses to indicate the less frequently sampled clade. Trees are scaled by number of substitutions per site.

*Figure S2*. Maximum likelihood trees derived from datasets of rabies virus sequences from Africa for a) a 405bp fragment of the nucleoprotein (N) gene (n=1397) with major African RABV clades indicated (Afr1/Cosmo: Africa 1/Cosmopolitan, Afr2: Africa2; Afr3: Africa 3, mongoose-associated clade; Afr4: Africa 4); and b) full length 1350bp nucleoprotein gene sequences (n=769) with the two Africa 1 subclades shown. Samples are coloured according to their country of origin as indicated on the map. All countries were sampled to at least partial N resolution. Trees are scaled by number of substitutions per site.

*Figure S3*. Maximum clade credibility trees from Bayesian phylogenetic estimation in BEAST for datasets of rabies virus sequences from the Africa 1B clade for increasing levels of genome coverage: a) a 405bp fragment of the nucleoprotein gene (n=510) from countries highlighted on the map, b) full 1350bp nucleoprotein gene (n=100) from the same countries except Botswana, Ghana, Kenya and Zimbabwe; and c) whole genome sequences from Tanzania. Trees are scaled by number of substitutions per site and diamonds indicate nodes with posterior probability support ≥0.9. Older samples from the Serengeti District (~20years old) are circled in the partial genome trees.

*Figure S4*. North-south phylogeographic structure among 60 rabies virus whole genome sequences isolated in Tanzania from 2003 to 2012. A maximum clade credibility tree is shown with branches coloured according to the most probable posterior location of their descendent nodes, inferred by discrete-state phylogeographic reconstruction using BEAST. The tree is scaled according to time in years and diamonds indicate node posterior support ≥0.9. The map and key indicate spatial division according to locations in the northern mainland (n=35), southern mainland (n=20) or Pemba island (n=5). Inset table provides details of dispersal pathways with Bayes Factor results and the estimated number of transitions according to Markov jumps counts, shown on the map with arrow width scaled by the number of transitions.
