# Supplementary material for: Elucidating the phylodynamics of endemic rabies virus in eastern Africa using whole-genome sequencing
Source: Virus Evol. 2015 Sep 10;1(1):vev011. doi: 10.1093/ve/vev011 (PMC5014479; doi:10.1093/ve/vev011)
Supplement: Supplementary Table S1 [file Supplementary_Material.docx]

**Supplementary text**

*Partial nucleoprotein (405bp) sequences*

An additional 50 partial N gene sequences were generated from samples obtained from Tanzania between 2004 and 2013 (Table S2). cDNA was prepared as described in the main text and a 405bp fragment amplified using hemi-nested PCR incorporating pan-*Lyssavirus* primers JW6UNI for first round products or JW10UNI for second round products, in combination with JW12, as previously described (1). Products were visualised on a 2% agarose gel with SYBR® Safe DNA Gel Stain (Invitrogen). First round positive PCR products were purified using a QIAquick PCR purification kit (Qiagen) and approximately 50–150 ng of product was used in a sequencing reaction with the Big Dye sequencing kit (Applied Biosystems). Sequencing was performed with the same primers on an ABI 3100 machine at the APHA sequencing facility and a consensus generated as previously described (1).

*Partial genome datasets*

Nucleoprotein sequences were retrieved from GenBank by searching for rabies virus records containing text matching Africa or any country in Africa. Only sequences with a length greater than 400bp were accepted and vaccines strains were excluded from the search. In addition, several datasets known to exist but which did not contain searchable text references were manually added to the search criteria (Box 1). Fasta files and GenBank records were downloaded in March 2015 and filtered to remove isolates not from Africa.

**Box 1. GenBank search for >400bp Rabies virus sequences from Africa**

((((((((((((((((((((((((((((((((((((((((((((((((((((((((((((((((((((((((((((((((((((((tanzania) OR africa) OR south africa) OR mozambique) OR zimbabwe) OR MAD) OR malawi) OR botswana) OR namibia) OR zambia) OR angola) OR congo) OR burundi) OR democratic republic of congo) OR burundi) OR rwanda) OR uganda) OR cameroon) OR gabon) OR central african republic) OR sudan) OR MAD[Text Word]) OR MOZ[Text Word]) OR NGA[Text Word]) OR zaire) OR madagascar) OR CAR) OR Mozambique) OR CAF) OR kenya) OR somalia) OR ethiopia) OR chad) OR nigeria) OR Nigeria) OR benin) OR togo) OR ghana) OR niger) OR mali) OR liberia) OR sierra leone) OR guinea) OR burkina faso) OR libya) OR mauritania) OR algeria) OR eritrea) OR egypt) OR morocco) OR tunisia) OR senegal) OR tanzania) OR Djibouti) OR Cote d'Ivoire) OR somalia) OR Guinea-Bissau) OR gambia) OR Republic of the Congo) OR Congo) OR Equatorial Guinea) OR ivory coast) OR swaziland) OR lesotho) OR Kissi[Author]) OR Talbi[Author]) AND rabies virus[Organism]) AND nucleoprotein[Title]) AND 400:11930[Sequence Length]) NOT turkey) NOT lebanon) NOT israel) NOT jordan))))))))) NOT rabies virus strain[Title])))))

*Additional whole genome sequencing protocols*

During the opimisation of protocols for whole genome sequencing some sequences used in the final analysis were generated by the following methods:

*i) Amplicon sequencing: 454 platform*

To generate cDNA 2μl of TRIzol-extracted viral RNA was subject to reverse transcription using the primer RABV_Tzdg.p1f (5′ ACGCTTAACAACAAAATCAGAG 3′) at a concentration of 2pmol/μl and Superscript III reverse transcriptase (Invitrogen) in a total volume of 20μl, as per manufacturer’s instructions. A working set of 26 short, tagged, overlapping primer pairs spanning the entire RABV genome was designed based on the full length Tanzanian dog reference genome RV2772 (accession: KF155002). Primers were used to obtain PCR products with 1µl of cDNA and a KOD hot start DNA polymerase kit as per manufacturer’s protocol (Novagen) with the following cycling parameters: 1 hold at 95 °C for 2 mins, 35 cycles at 95 °C for 20 s, 50-60°C (dependent on the optimized temperature for each primer pair) for 20 s, 70 °C for 20 s and a final hold at 70 °C for 10 min using a 2720 thermal cycler (Applied Biosystems). Products were pooled for each sample and sent to the APHA sequencing facility for template preparation and 454 pyrosequencing.

*ii) Depleted RNA: 454 platform*

TRIzol-extracted viral RNA was depleted of host genomic DNA and ribosomal RNA as described in the main text. Random-primed cDNA libraries were constructed and sent to the APHA sequencing facility for 454 pyrosequencing.

*iii) Depleted RNA: Illumina NextSeq™ 500 platform*

Double stranded cDNA was synthesized as described in the main text and sent to the Glasgow Polyomics facility (University of Glasgow, Glasgow, UK) for Nextera XT library preparation and sequencing on an Illumina NextSeq™ 500 platform.

1. Heaton PR, Johnstone P, McElhinney LM, Cowley R, O’Sullivan E, Whitby JE. Heminested PCR assay for detection of six genotypes of rabies and rabies-related viruses. J Clin Microbiol. 1997;
